# Comparison of Time–Frequency Characteristics of Lower Limb EMG Signals Among Different Foot Strike Patterns During Running Using the EEMD Algorithm

**DOI:** 10.3390/life15091386

**Published:** 2025-09-01

**Authors:** Shuqiong Shi, Xindi Ni, Loi Ieong, Lei Li, Ye Liu

**Affiliations:** 1 School of Sport Science, Beijing Sport University, Beijing 100084, China; 2019112024@bsu.edu.cn (S.S.); nxd_bsu@foxmail.com (X.N.); loi_326@hotmail.com (L.I.); lilei120800@163.com (L.L.); 2 Key Laboratory of Exercise and Physical Fitness of Ministry of Education, Beijing 100084, China; 3 Laboratory of Sports Stress and Adaptation of General Administration of Sport, Beijing Sport University, Beijing 100084, China

**Keywords:** running, time–frequency analysis, empirical mode decomposition, sports biomechanics

## Abstract

Runners have a high probability of sports injuries due to improper landing patterns. This study aimed to investigate the effects of three different foot strike patterns on lower limb muscle activation in healthy young male university students without specialized sports training experience. Methods: Sixteen healthy male college students (age: 21 ± 1 years) participated in this study. They performed running with three different foot strike patterns: forefoot strike (FFS), midfoot strike (MFS), and rearfoot strike (RFS) at controlled speeds of 1.4–1.6 m/s. EMG signals from six lower limb muscles (vastus lateralis, vastus medialis, rectus femoris, tibialis anterior, lateral gastrocnemius, and medial gastrocnemius) during the stance phase were collected using a wireless EMG system (1000 Hz). Ensemble Empirical Mode Decomposition (EEMD) was employed to analyze the time–frequency characteristics of lower limb EMG signals and ankle joint co-activation patterns to investigate the corresponding neuromuscular control mechanisms. Statistical analyses were performed using repeated-measures ANOVA, and significance was set at *p* < 0.05. Results: The timing of maximum energy in lower limb muscles during the stance phase occurred earlier in RFS compared to FFS and MFS. At initial ground contact, the low-frequency component energy (below 60 Hz) of the medial gastrocnemius was significantly higher in MFS and RFS compared to FFS, while FFS exhibited significantly higher high-frequency component energy (61–200 Hz). The co-activation of ankle dorsiflexors and plantar flexors (TA/GM) was also significantly higher in MFS and RFS compared to FFS. During the 100 ms before foot contact, the low-frequency component energy (below 60 Hz) of the lateral gastrocnemius was significantly higher in MFS compared to FFS, and the degree of TA/GM co-activation was significantly higher in both MFS and RFS compared to FFS. Conclusions: The maximum frequency in lower limb muscles appeared earliest during the mid-stance phase in the rearfoot strike (RFS) pattern. Moreover, during the pre-activation and early stance phases, frequency differences were observed only in the medial gastrocnemius, with RFS showing significantly higher low-frequency power.

## 1. Introduction

Running, as a common form of exercise, has positive effects on promoting cardiovascular health and is one of the preferred activities for people pursuing health. However, it also carries a high risk of sports injuries. Research indicates that distance running causes high rates of running injuries, with estimates ranging from 30 to 75% per year among various runner populations, and injury rates appear to remain persistently high despite technological advances in footwear [1,2]. Based on the position of foot contact with the ground at the moment of landing, running foot strike patterns can be categorized into three types: forefoot strike (FFS), midfoot strike (MFS), and rearfoot strike (RFS). During running, foot strike patterns significantly influence the specific biomechanical loads experienced by lower limb structures and tissues throughout the running process [3]. Previous studies have indicated that different foot strike patterns during running lead to changes in impact frequencies experienced by the human body, while also affecting the degree and timing of muscle activation [4]. Some studies investigating variability in human running [2,5,6,7,8] have found that the instant of foot contact is the primary source of running variability [7], which may reflect neuromuscular system adjustments and adaptations to each independent and distinct landing moment. These adjustments and adaptations typically involve coordinated muscle actions around joints, where simultaneous contraction of agonist and antagonist muscles (i.e., co-activation) affects movement efficiency [9] and joint stability [10], serving as an important indicator of neuromuscular control. Therefore, in-depth understanding of neuromuscular control mechanisms under different foot strike patterns holds significant practical implications for preventing running injuries and optimizing training protocols.

Electromyography (EMG) is widely used to assess muscle activity and function. While conventional time-domain analysis provides useful information about muscle coordination, it cannot fully capture the complex neural control involved in dynamic movements. Time–frequency analysis methods, such as short-time Fourier transform (STFT) and wavelet transform, have thus been applied to EMG to investigate muscle activation patterns and changes in timing and intensity under various conditions [11,12,13,14]. However, these linear methods rely on fixed basis functions and often face limitations in resolving the nonlinear and non-stationary characteristics typical of biomedical signals [14,15]. As a result, they may not accurately separate overlapping signal components or adapt to the rapidly changing features of EMG during dynamic tasks.

To address these limitations, Huang et al. [16] proposed empirical mode decomposition (EMD), an adaptive, data-driven approach capable of decomposing nonlinear and non-stationary signals into intrinsic mode functions (IMFs). EMD is better suited to capture the aperiodic and unstable nature of muscle activity [17], but its effectiveness is reduced by sensitivity to noise and mode mixing. Ensemble empirical mode decomposition (EEMD) improves upon EMD by introducing white noise to eliminate mode mixing, resulting in more stable and reliable signal decomposition [18]. Studies have shown that EEMD-based methods provide superior performance compared to traditional digital filters and linear time–frequency techniques in removing noise and analyzing muscle fatigue, motor control, and rehabilitation progress [19,20,21,22,23,24]. Therefore, EEMD offers unique advantages for exploring the detailed time–frequency characteristics of EMG signals during complex movement patterns, making it especially suitable for the current study.

Previous research has explored the effects of different foot strike patterns on impact frequencies experienced by the human body; however, the underlying neuromuscular control mechanisms-especially how the nervous system coordinates lower limb muscles in response to these impacts—remain unclear, particularly in recreational runners. To minimize potential confounding effects from prior sports-specific training on neuromuscular control and muscle activation patterns, this study specifically recruited healthy male college students without specialized athletic training. Therefore, this study applies the ensemble empirical mode decomposition (EEMD) algorithm to analyze the time–frequency characteristics of lower limb EMG signals before and after foot contact across three foot strike patterns. By combining advanced nonlinear signal analysis with co-activation indices, our approach provides new insights into how foot strike patterns influence muscle coordination and joint stability. To our knowledge, this is the first exploratory study to apply EEMD-based time–frequency analysis to investigate neuromuscular adaptations across different foot strike patterns in a population without specialized sports training. These findings enhance our understanding of neuromuscular adaptation during running and offer practical guidance for injury prevention and training in the general running population.

Therefore, we hypothesized that there would be significant differences in the time–frequency characteristics of lower limb muscle activation and ankle joint muscle co-activation among the three foot strike patterns during running.

## 2. Materials and Methods

### 2.1. Subjects

Seventeen healthy male college students from a university were initially recruited for this study. Inclusion criteria were: no specialized sports training experience, no lower limb joint or muscle injuries within the past three months, normal range of motion in all joints, no recent high-intensity exercise, good rest the day before testing, and voluntary participation with commitment to complete the experiment. After screening, 16 participants were finally included (age: 21 ± 1 years; height: 177 ± 6 cm; body weight: 76.15 ± 13.46 kg). Leg dominance was assessed by asking each participant which leg they would use to kick a ball; all participants reported right leg dominance. Therefore, all measurements were performed on the right leg.

All participants reported no regular running training or competitive running background, and their weekly running mileage was less than 5 km, indicating limited recreational running experience. To minimize potential confounding factors, all tests were conducted during the daytime to reduce the influence of circadian rhythms. Participants were instructed to avoid caffeine and strenuous physical activity for at least 24 h before testing, and to maintain their usual diet and have a light meal at least 2 h prior to the experiment. They also confirmed adequate sleep (at least 7 h) the night before testing and no subjective fatigue on the day of the test.

An a priori power analysis was conducted using G*Power 3.1.9.7 to determine the required sample size for the repeated measures design (MANOVA, within factors). The analysis indicated that, given an effect size f = 0.8, α = 0.05, and power = 0.95, a minimum of 12 participants was required. Thus, the final sample size met the statistical power requirements. Importantly, our within-subject (repeated measures) design, in which each participant completed three test sessions, helps control for inter-individual variability and increases statistical power. As noted by Hopkins [25], repeated measures designs can substantially reduce the required sample size compared to between-subjects designs. Our sample size is also consistent with field recommendations [26] and aligns with recent studies in the area (e.g., Mesquida et al., 2023 [27]).

### 2.2. Experimental Methods

Prior to the experimental trials, each participant completed a standardized warm-up consisting of 5 min of light jogging followed by dynamic stretching exercises for the lower limbs. All running trials were conducted on a flat, outdoor 400-meter standard track under stable weather conditions. After warm-up, experimental subjects performed running with three different foot strike patterns at their natural cadence: FFS (forefoot contacts the ground first at the moment of landing), MFS (both forefoot and rearfoot participate in ground contact at the moment of landing), and RFS (heel contacts the ground first at the moment of landing). For each foot strike pattern, participants completed three valid trials, each covering a distance of 10 m at a controlled speed of 1.4–1.6 m/s, with at least 1 min of rest between each trial to minimize fatigue. Running speed was monitored using a speed meter (Smartspeed, Fusion Sport, Brisbane, QLD, Australia). Prior to testing, a force platform (KISTLER-3D, sampling frequency 1000 Hz) was placed flat on the floor with extended runways at both ends to record ground reaction forces (GRFs) during the stance phase of running. Simultaneously, a Trigno™ wireless muscle activity acquisition system (DELSYS Trigno Wireless EMG System, sampling frequency 1000 Hz) was used to collect EMG activity during the stance phase from six muscles of the subjects’ right leg (all subjects were right-leg dominant): vastus lateralis (VL), vastus medialis (VM), rectus femoris (RF), tibialis anterior (TA), gastrocnemius lateralis (GL), and gastrocnemius medialis (GM). Data collection was synchronized with the force platform through a synchronization trigger box.

Before testing, experimental subjects were required to change into standardized athletic shoes, socks, and shorts. Skin preparation at the electrode sites was performed using alcohol to reduce impedance. Surface EMG sensors were then attached to the target muscles of the right leg following the SENIAM guidelines [28] to ensure accurate and reproducible placement. Subsequently, subjects practiced running with FFS, MFS, and RFS patterns until they could run naturally on the runway with each pattern. The starting point was adjusted according to the force platform position and each subject’s stride length, ensuring that subjects could naturally run over the force platform with their right foot landing precisely on the platform, avoiding deliberate targeting of the platform. If deliberate targeting occurred, the starting position was readjusted and measurements repeated. A high-speed camera (HIKROBOT, MV-CU020-90GC, sampling frequency 60 Hz) was used to record running movements from the subject’s lateral view to identify different foot strike patterns. RFS was defined as initial foot–ground contact occurring only at the heel or posterior third of the foot, with no contact from the midfoot or forefoot at the moment of landing. MFS was defined as initial foot–ground contact involving not only the posterior third of the foot but also the midfoot or entire foot sole. FFS was defined as initial foot–ground contact occurring at the forefoot or anterior half of the foot, with no heel contact at the moment of landing [29]. For each experimental subject, the three foot strike patterns were performed in random order to avoid the influence of testing sequence on experimental data, with three valid trials conducted for each running pattern.

### 2.3. Data Analysis

Raw EMG signals were processed in MATLAB 2022a (Mathworks, Inc., Natick, MA, USA). For noise reduction, a fourth-order zero-phase Butterworth band-pass filter was applied with cut-off frequencies of 10–450 Hz. The filtered EMG signals were then full-wave rectified and smoothed using a moving RMS window of 50 ms. Each muscle’s EMG amplitude was normalized to the mean of the peak RMS values obtained from three RFS stance phases for each subject, consistent with previous studies [30].

Empirical mode decomposition (EMD) was employed to process the filtered EMG signals. EMD is an adaptive time–frequency analysis method that decomposes a time series signal S(t), 1≤t≤L, into the sum of multiple intrinsic mode functions (IMFs) $C_{i}(t)$, 1≤i≤N,

(1) $$S\left(t\right)=\sum_{i=1}^{N}C_{i}\left(t\right)+T_{N}\left(t\right)$$

where t is time, N is the number of IMFs, and $r_{N}(t)$ is the residual signal [16]. An IMF is a zero-mean amplitude-modulated (AM) and frequency-modulated (FM) signal that must satisfy two conditions: (1) the number of extrema (including local maxima and minima) and zero-crossing points in the time series differs by at most one; (2) throughout the entire time series, the mean of the upper envelope (defined by maxima) and lower envelope (defined by minima) is zero. Therefore, an IMF is a simple oscillatory mode that is symmetric about zero.

The decomposition technique for sequentially extracting each IMF from the original signal is called the sifting process. The process begins by identifying local maxima and minima of x(t), where x(t) is an auxiliary variable of the original signal S(t). Two cubic spline curves are fitted to the local maxima and minima, forming the upper envelope *E_u_* and lower envelope *E_l_*, respectively. Subtracting the mean of $E_{u}$ and $E_{l}$ (i.e., $E_{m}$) from x(t) yields a new signal x(t). The above steps are iteratively executed until x(t) is updated to an IMF $C_{1}(t)$ that satisfies the two defining conditions. The residual signal $r_{1}(t)$ is calculated by subtracting the first IMF $C_{1}(t)$ from the initial x(t), then $r_{1}(t)$ is treated as a new signal, and this sifting process is repeated to obtain all subsequent IMFs that satisfy the conditions, until the residual $r_{N}(t)$ becomes a constant, monotonic slope, or a function with only one extremum. If the residual $r_{N}(t)$ is denoted as the (N+1)th order IMF $C_{N+1}(t)$, its expression can be written as follows:

(2) $$S\left(t\right)=\sum_{i=1}^{N+1}C_{i}\left(t\right)$$

After the above sifting process, the original signal yields low-order IMFs containing fast oscillatory modes, while high-order IMFs contain slow oscillatory modes [16].

Ensemble empirical mode decomposition (EEMD) is a noise-assisted method for optimizing EMD, where the sifting process is performed on a series of noise-added signals u(t), each obtained by adding the original signal S(t) to different finite-amplitude white noise W(t), i.e., $u\left(t\right)=S\left(t\right)+W(t)$. Each u(t) can be decomposed using EMD. The resulting IMFs, denoted as $C_{ij}(t)$, are obtained by averaging all IMFs obtained from iterations, with the following expression:

(3) $$C_{i}\left(t\right)=\frac{1}{N_{T}}\sum_{j=1}^{N_{T}}C_{ij}\left(t\right)$$

where i is the IMF order, j is the iteration index, and $N_{T}$ is the total number of iterations.

Adding white noise promotes the final IMFs to be relatively independent of the signal’s local temporal characteristics on comparable scales, thereby reducing mode mixing caused by conventional EMD. This method has been proven to significantly reduce the total power of EMG noise while preserving most of the signal energy [31], facilitating nonlinear analysis of non-stationary EMG signals.

The detailed parameters for EEMD implementation are as follows: EEMD was implemented using a custom MATLAB script based on Wu and Huang [16]. The amplitude of the added white noise was set to 0.2 times the standard deviation of the original EMG signal (Nstd = 0.2). The ensemble number was set to 100 (NE = 100), and the number of sifting iterations for each IMF extraction was set to 10.

For a given time series signal S(t), its Hilbert transform (HT) y(t) is as follows:

(4) $$y\left(t\right)=\frac{1}{\pi }P\int_{-\infty }^{\infty }\frac{S(\tau )}{t-\tau }d\tau$$

where P is the Cauchy principal value.

Let z(t) be the associated analytic signal of $z\left(t\right)=S\left(t\right)+iy\left(t\right)=a\left(t\right)Exp(i\theta \left(t\right))$, where $a\left(t\right)=\sqrt{S^{2}\left(t\right)+y^{2}(t)}$ is the instantaneous amplitude; $\theta \left(t\right)=arctan(y(t)/S(t))$ is the instantaneous phase associated with signal S(t). The instantaneous angular frequency ω(t) is dθ(t)/dt.

Applying HT to each IMF component obtained from EMD signal decomposition, signal S(t) can be expressed as the real part of z(t):

(5) $$S(t)=Re[\sum_{m=1}^{M}a_{m}\left(t\right)E(i\int_{-\infty }^{\infty }\omega_{m}\left(t\right)dt)]$$

Equation (5) can be considered as a generalized nonlinear and non-stationary form of Fourier decomposition of S(t), where the instantaneous amplitude and frequency of each IMF are functions of time in three-dimensional or two-dimensional plots. In three-dimensional plots, amplitude (or energy) can be plotted as contours on the instantaneous frequency-time plane with *z*-axis surfaces. In two-dimensional plots, color scales can be used to represent amplitude or energy in the instantaneous frequency-time plane. This plot in the instantaneous frequency-time plane is called the Hilbert spectrum (HS) H(ω,t). The marginal Hilbert spectrum (MHS) can be defined based on HS H(ω,t) as follows:

(6) $$h\left(\omega \right)=\int_{0}^{T}H\left(\omega ,t\right)dt$$

MHS provides a measure of the total energy (or amplitude) contribution of each frequency value over the entire data time interval [0,T], thereby obtaining instantaneous frequency and instantaneous energy, and calculating the stance phase timing (S_max_) and frequency (F_max_) corresponding to maximum energy for all six muscles during the entire stance phase.

The total signal power (P_sum_) for two frequency bands (below 60 Hz and 61–200 Hz) during the 100 ms before foot contact, the first 20% of the stance phase, and the 20~40% stance phase, denoted as P_60Hz_ and P_60–200Hz_.

After removing the residual signal obtained from EMD decomposition of EMG signals and reconstructing the EMG signals, the root mean square (RMS) of the six muscles during the stance phase was calculated with a window width of 50 ms. The RMS of lower limb muscles was normalized against the average maximum values from three trials during the RFS running stance phase. Stance phase time was also normalized.

Finally, the co-activation index (CI, co-contraction index) of ankle flexor and extensor muscles during the 100 ms before foot contact, the first 20% of the stance phase, and the 20~40% stance phase was calculated using Equation (7). Co-activation refers to the degree of simultaneous contraction and activation of multiple muscles, where >100% indicates greater antagonist muscle activation, and <100% indicates greater agonist muscle activation [32].

(7) $$CI=\frac{2\cdot {EMG}_{TA}}{{EMG}_{GM}+{EMG}_{TA}}*100$$

### 2.4. Statistical Analysis

All experimental data were processed using SPSS 18.0 for statistical analysis, with results expressed as mean ± standard deviation. The normality of the data was assessed using the Shapiro-Wilk test, and homogeneity of variance was evaluated with Mauchly’s test of sphericity. One-way repeated measures analysis of variance and post hoc multiple comparison tests were used to examine differences in RMS, S_max_, F_max_, P_60Hz_, and P_60–200Hz_ among different foot strike patterns. When significant main effects were found, the least significant difference (LSD) test was used for post hoc multiple comparisons. Statistical significance was set at *p* < 0.05. All results are presented as mean ± standard deviation (SD).

## 3. Results

**Figure 1:**
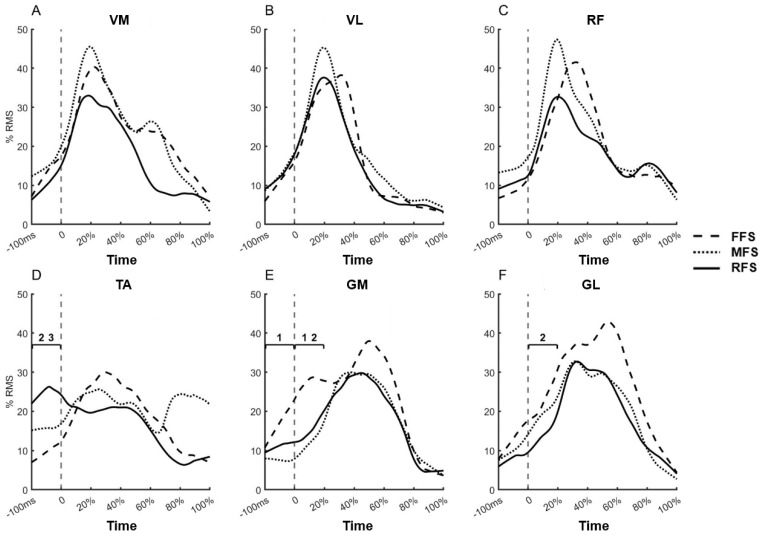
Electromyographic (EMG) activity (% RMS) of the VM (**A**), VL (**B**), RF (**C**), TA (**D**), GM (**E**), and GL (**F**) muscles during the stance phase for forefoot strike (FFS, dashed line), midfoot strike (MFS, dotted line), and rearfoot strike (RFS, solid line) running patterns. Numbers indicate significant differences between foot strike patterns (*p* < 0.05): 1, FFS vs. MFS; 2, FFS vs. RFS; 3, MFS vs. RFS. The *x*-axis represents time, with 0 ms indicating initial foot contact (touchdown), −100 ms representing 100 ms before touchdown, and 0–100% denoting the normalized stance phase. The vertical dashed line indicates the time of initial foot contact (0 ms).

In terms of time-domain analysis, there were no significant differences in the average RMS of lower limb muscles during the stance phase when running with the three different foot strike patterns (Table 1). During the 100 ms before foot contact, the RMS of tibialis anterior (TA) was significantly higher in RFS compared to FFS and MFS (Figure 1); the RMS of gastrocnemius medialis (GM) was significantly higher in FFS compared to MFS (Figure 1). During the 0~20% phase of stance, the RMS of GM was significantly higher in FFS compared to MFS and RFS (*p* < 0.05) (Figure 1); the RMS of gastrocnemius lateralis (GL) was also significantly higher in FFS compared to MFS (*p* < 0.05) (Figure 1). The specific *p*-values and effect sizes are presented in Appendix A (Table A1).

**Figure 2:**
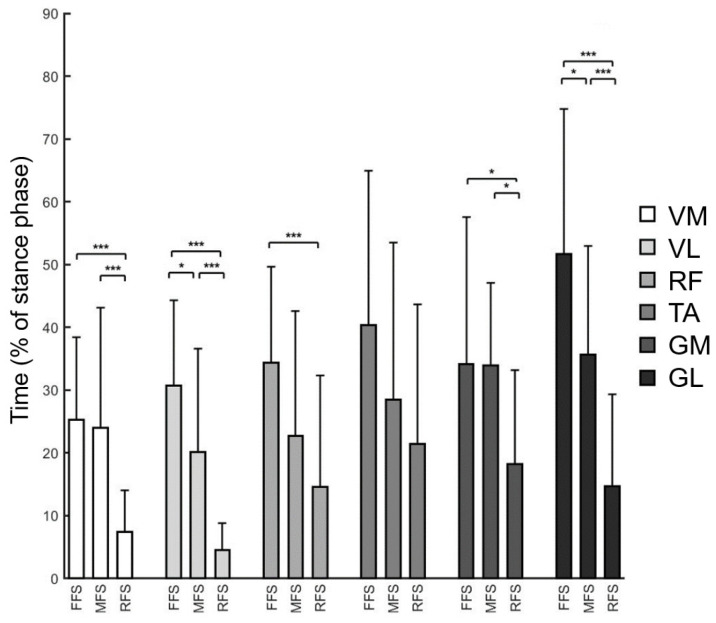
Time point (mean ± SD) corresponding to the maximum energy in the Hilbert–Huang Transform (HHT) time–frequency matrix of EMG signals, calculated using the EEMD algorithm, for the VM, VL, RF, TA, GM, and GL muscles during the stance phase in running with three foot strike patterns: forefoot strike (FFS), midfoot strike (MFS), and rearfoot strike (RFS). The *y*-axis represents the time of maximum energy as a percentage of the stance phase (% of stance phase). Data are presented as mean ± standard deviation (SD). Error bars represent the SD. Significant differences between groups are indicated as follows: * *p* < 0.05; *** *p* < 0.001.

In terms of time–frequency analysis, Smax of VM, VL, GM, and GL appeared significantly earlier in RFS compared to FFS and MFS (*p* < 0.05; Figure 2). However, no significant differences were observed in Fmax of lower limb muscles among the three foot strike patterns (Table 2). The specific *p*-values and effect sizes are presented in Appendix A (Table A2).

**Figure 3:**
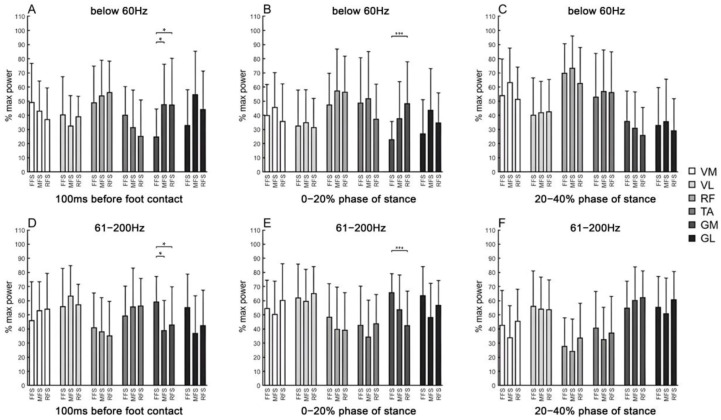
Normalized EMG signal power (mean ± SD) of lower limb muscles within two frequency bands (below 60 Hz and 61–200 Hz) during three phases of running with different foot strike patterns (FFS, MFS, RFS): 100 ms before foot contact (**A**,**D**), 0–20% of the stance phase (**B**,**E**), and 20–40% of the stance phase (**C**,**F**). Data are presented as mean ± standard deviation (SD). Error bars represent the SD. Signal power is expressed as a percentage of the maximal value (% of maximal power). Asterisks indicate significant differences between groups (* *p* < 0.05; *** *p* < 0.001).

Regarding the frequency-domain analysis, the normalized EMG signal power of lower limb muscles in both low (below 60 Hz) and high (61–200 Hz) frequency bands was compared among the three foot strike patterns (FFS, MFS, RFS) during different phases of running. Significant differences were observed in certain muscles and time windows (Figure 3). The specific *p*-values and effect sizes are presented in Appendix A (Table A3).

## 4. Discussion

In this study, we investigated the time–frequency characteristics and co-activation patterns of lower limb muscles during running with different foot strike patterns (forefoot, midfoot, and rearfoot strikes). Our main findings showed significant differences in muscle activation and coordination, particularly in the gastrocnemius and tibialis anterior, across the three patterns during both the initial contact and pre-activation phases.

The comparative results of lower limb muscle activity during running with forefoot, midfoot, and rearfoot strike patterns in this study show that the RMS of gastrocnemius muscles was significantly higher in FFS compared to RFS during the initial contact phase (0–20%) of running, which is consistent with previous research findings [33,34,35]; while during the 100 ms before foot contact, the RMS of tibialis anterior was significantly higher in RFS compared to FFS. However, further comparison of the time–frequency information of lower limb muscle activity revealed differences in the stance phase timing corresponding to maximum muscle energy (S_max_) (Figure 2). The S_max_ of lower limb muscles, primarily VM, VL, GM, and GL, occurred earlier during RFS running and mainly appeared in the early and middle phases of stance. However, the frequency corresponding to maximum energy (60–250 Hz) showed no significant differences when comparing FFS and RFS (Table 2). A study on time–frequency analysis of vertical ground reaction forces during running with different foot strike patterns found that the impact frequency during initial contact was significantly higher when using RFS running [4], which corresponds to the results of this study. It is speculated that the earlier occurrence of S_max_ in lower limb muscles during RFS running may serve to buffer the higher frequency impacts received during landing, thereby avoiding resonance in soft tissues. This suggests that lower limb muscles may adjust their activation frequency to respond to the impact frequency experienced during landing when running with different foot strike patterns.

Research suggests that time–frequency analysis of electromyographic signals can reveal muscle activation patterns during running, which consist of two components: general muscle activation patterns and refined muscle activation patterns [12]. The general muscle activation pattern represents the initiation and termination of muscle force generation, providing the majority of force required for movement, and can be reflected by the power of high-frequency components in EMG signals [12]. EMG-related simulation studies have also confirmed a positive correlation between high-frequency component power and the number of motor unit action potentials (MUAPs) [36]. Some research has attempted to link high-frequency power with muscle fiber types [37], but this association has been questioned due to lack of verification [38].

During the 0–20% phase of stance, the gastrocnemius muscle in FFS demonstrated significantly higher activation levels (Figure 1), which is consistent with previous studies [33,34,35]. Researchers suggest this increased activation serves to enhance ankle joint function for attenuating ground impact forces. The ankle joint maintains substantial plantarflexion at initial contact to store elastic potential energy and absorb impact forces through muscle work and joint mechanics. Furthermore, during this phase, the GM P_60–200Hz_ in FFS was significantly higher than in RFS, with all these results indicating greater ankle plantar-flexor force generation.

Combined with our previous research findings [4], FFS running is associated with lower impact frequency and reduced lower limb stiffness, which is unfavorable for elastic energy storage and utilization in the lower extremities. We hypothesize that during FFS running, because the ground reaction force acts anterior to the ankle joint, creating a tendency for ankle dorsiflexion, greater recruitment of large motor units is required to generate relatively greater ankle plantarflexor force, manifesting as increased gastrocnemius activation to maintain FFS running mechanics. This also supports previous research conclusions that FFS running increases gastrocnemius work output, subsequently increasing Achilles tendon loading. Therefore, FFS running requires enhanced eccentric working capacity of the gastrocnemius muscle to prevent injury occurrence [34,39]. Therefore, runners who wish to adopt or maintain a forefoot strike pattern should incorporate specific eccentric strengthening exercises for the gastrocnemius during training to reduce the risk of injury.

Furthermore, during the 0–20% phase of stance, the low-frequency component signal power (P_60Hz_) of GM in RFS was significantly higher than in FFS. During movement, the refined muscle activation pattern primarily manifests as the degree of synchronization of motor unit action potentials, which can be reflected by the power of low-frequency components in EMG signals. Piper waves in EMG signals are considered to reflect refined muscle activation patterns during movement, and the use of nonlinear methods to analyze the power of β (8–25 Hz) and low γ (25–70 Hz) frequency bands (Piper waves) in EMG signals has been confirmed [11]. Research by Von Tscharner et al. has demonstrated that Piper waves can be observed in GM and GL [12], as well as VM and VL [40], during each gait cycle of human running. They suggest that this EMG rhythm reflects fine-tuning after muscle activation, representing adjustments to applied forces and modifications of preferred, pre-programmed activation patterns in response to external changes [41].

Additionally, consistent with Valencia et al.’s findings [34], this study found that the percentage of ankle joint flexor-extensor co-activation in RFS was significantly greater than in both FFS and RFS. The degree of co-activation between antagonist and agonist muscles around a joint can reflect joint stiffness and shows a positive correlation, meaning higher co-activation levels correspond to greater joint stiffness [42]. This study suggests that during RFS running, ground reaction forces are transmitted upward from the calcaneus along the long axis of the tibia, creating a tendency for ankle plantarflexion. Under these conditions, the GM increases its synchronization degree to enhance muscle force generation within a short time period, maintaining higher ankle joint stiffness, thereby demonstrating higher low-frequency component energy and TA/GM co-activation levels.

Different landing patterns also influence the pre-activation of lower limb muscles. During the 100 ms before foot contact, RFS demonstrated significantly higher TA activation levels, while FFS showed higher gastrocnemius activation levels (Figure 1). Simultaneously, the co-activation of ankle flexor-extensor muscles in RFS approached 100% (Table 3). 100% co-activation is considered ideal antagonist-agonist synergistic contraction [32], and the results of this study indicate that this is primarily attributed to increased TA activation levels in RFS. From an energy utilization perspective, achieving high co-activation levels before ground contact appears to be energy wasteful, as simultaneous activation of agonist and antagonist muscles requires energy expenditure that does not contribute to the task [42]. A study by Tam [43] on running economy in experienced runners found that pre-activation levels before landing were negatively correlated with running economy, suggesting that increased pre-activation prior to ground contact may actually increase the metabolic cost and oxygen consumption during running. In contrast, our current findings indicate that higher activation levels before ground contact, particularly increased TA/GM co-activation, may play a role in regulating ankle joint stiffness, potentially reducing energy expenditure after ground contact by optimizing force transmission and stability at initial contact.

Furthermore, the low-frequency component signal power (P_60Hz_) of GM in RFS was also significantly greater. Both findings suggest that during the pre-activation period of ankle flexor-extensor muscles before ground contact in RFS, ideal synergistic contraction and synchronization are demonstrated, which can contribute to the subsequent greater co-activation levels during the early stance phase, assisting the ankle joint in maintaining greater stiffness and stability. This is beneficial for increasing gastrocnemius energy absorption and utilization at the moment of ground contact, achieving higher running economy, and supporting the author’s previous research conclusions [4]. Such insights can assist coaches and clinicians in developing training strategies aimed at improving running economy through muscle activation monitoring and adjustment. However, this study only compared different landing patterns in subjects without long-distance running training experience. Future research should further compare differences in experienced runners to explore these findings more thoroughly. In addition, anatomical and biomechanical factors such as individual differences in tendon properties, joint alignment, or foot structure may also contribute to the observed results. These factors were not directly assessed in this study and should be considered in future research to better understand their potential influence on lower limb muscle activation patterns during different foot strike patterns.

### Limitations

The sample size in this study was calculated based on a large effect size (f = 0.8), which ensures sufficient power to detect large effects. However, as this is an exploratory study with a relatively small sample, smaller or moderate effects may not have been detected due to limited statistical power. Future research should consider increasing the sample size to enhance the robustness and generalizability of the findings. In addition, the study specifically recruited healthy young male participants without specialized sports training to minimize the confounding effects of sex and training experience, focusing on neuromuscular differences among different foot strike patterns. Nevertheless, sex and training history are known to influence neuromuscular control and running biomechanics, which could affect foot strike patterns. Therefore, the generalizability of our findings is limited, and future studies should include female participants and individuals with varying levels of running experience. Finally, the relatively slow running speed used in this study was chosen for participant comfort and compliance, but higher running speeds may influence neuromuscular activation patterns and differences among foot strike patterns. Future research should explore these effects at different running speeds.

## 5. Conclusions

This study used the EEMD algorithm to analyze lower limb EMG signals during the stance phase of running with different foot strike patterns. We found that in the rearfoot strike (RFS) pattern, the maximum muscle frequency occurred earliest during mid-stance, and RFS was associated with significantly higher low-frequency power in the medial gastrocnemius during pre-activation and early stance. These findings highlight the key role of the calf muscles, particularly the medial gastrocnemius, in adapting to different foot strike techniques, especially during pre-activation and ground contact.

Practically, our results suggest that runners and coaches should pay particular attention to calf muscle conditioning and activation strategies when considering or training different foot strike patterns. Future studies should include participants with long-distance running experience and assess additional biomechanical factors to further validate and generalize these findings.

## Figures and Tables

**Table 1:** The average muscle RMS of the lower limb muscles during the stance phase of running under three different foot strike patterns (data are expressed as mean ± SD).

	VM	VL	RF	TA	GM	GL
FFS	30.80% ± 20.26%	24.57% ± 14.34%	25.51% ± 14.54%	35.51% ± 21.22%	35.32% ± 17.18%	42.82% ± 17.35%
MFS	30.81% ± 14.91%	22.38% ± 11.26%	25.60% ± 13.09%	40.07% ± 49.71%	25.87% ± 11.72%	31.87% ± 8.34%
RFS	24.19% ± 5.91%	22.33% ± 5.38%	25.80% ± 8.17%	28.41% ± 6.78%	25.78% ± 5.63%	29.30% ± 8.20%

**Table 2:** The instantaneous frequency corresponding to the maximum energy of the lower limb muscles during the foot stance phase of running under three different foot strike patterns (data are expressed as mean ± SD).

	VM	VL	RF	TA	GM	GL
FFS	96.56 ± 27.17	92.25 ± 28.31	69.13 ± 25.36	106.00 ± 44.70	140.69 ± 44.26	121.88 ± 59.61
MFS	78.31 ± 36.98	88.56 ± 34.02	69.50 ± 31.20	90.19 ± 55.75	128.19 ± 38.81	124.94 ± 54.40
RFS	88.00 ± 35.08	95.88 ± 38.72	66.56 ± 28.74	101.13 ± 49.31	122.25 ± 39.09	126.44 ± 50.03

**Table 3:** Co-activation (%) of the ankle dorsiflexor and plantar flexor muscles (data are expressed as mean ± SD).

	100 ms Before Foot Contact	0–20% Phase of Stance	20–40% Phase of Stance
FFS	16.50 ± 11.35 ^1,2^	88.28 ± 40.01 ^1,2^	101.18 ± 39.18
MFS	79.74 ± 37.04	128.33 ± 44.23	99.32 ± 39.29
RFS	101.00 ± 19.90	134.08 ± 47.95	84.18 ± 25.39

^1^ indicates a significant difference between FFS and MFS (*p* < 0.05); ^2^ indicates a significant difference between FFS and RFS (*p* < 0.05).

## Data Availability

The data presented in this study are available on request from the corresponding author. The data are not publicly available due to privacy and ethical restrictions, as they contain information that could compromise the privacy of research participants.

## Abbreviations

The following abbreviations are used in this manuscript:

EMD	Empirical mode decomposition
EEMD	Ensemble empirical mode decomposition
FFS	Forefoot strike
MFS	Midfoot strike
RFS	Rearfoot strike
VM	Vastus medialis
VL	Vastus lateralis
RF	Rectus femoris
TA	Tibialis anterior
GM	Gastrocnemius medialis
GL	Gastrocnemius lateralis

## Appendix A

**Table A1:** ES for muscle EMG activity comparison among foot strike patterns during the stance phase (related to Figure 1).

Musle	Phase	*p*	η^2^	Cohens’ d (*p* Value)		
				FFS vs. MFS	FFS vs. RFS	MFS vs. RFS
VM	Before Strike	0.57	0.02	0.08 (0.95)	0.31 (0.74)	0.38 (0.56)
VM	0–20%	0.30	0.05	0.37 (0.47)	0.11 (0.95)	0.52 (0.31)
VM	20–40%	0.29	0.05	0.08 (0.96)	0.42 (0.45)	0.64 (0.30)
VM	40–60%	0.79	0.01	0.03 (0.99)	0.22 (0.80)	0.21 (0.86)
VM	60–80%	0.28	0.05	0.00 (1.00)	0.53 (0.34)	0.56 (0.35)
VM	80–100%	0.57	0.02	0.22 (0.77)	0.33 (0.55)	0.15 (0.94)
VL	Before Strike	0.83	0.01	0.20 (0.82)	0.12 (0.94)	0.09 (0.96)
VL	0–20%	0.49	0.03	0.34 (0.48)	0.13 (0.95)	0.30 (0.66)
VL	20–40%	0.84	0.01	0.04 (0.99)	0.26 (0.84)	0.14 (0.91)
VL	40–60%	0.73	0.01	0.02 (1.00)	0.29 (0.79)	0.24 (0.75)
VL	60–80%	0.47	0.03	0.20 (0.76)	0.32 (0.86)	0.38 (0.44)
VL	80–100%	0.80	0.01	0.22 (0.78)	0.14 (0.95)	0.11 (0.93)
RF	Before Strike	0.46	0.03	0.37 (0.43)	0.43 (0.86)	0.21 (0.75)
RF	0–20%	0.15	0.07	0.56 (0.15)	0.34 (0.88)	0.41 (0.34)
RF	20–40%	0.48	0.03	0.04 (0.99)	0.46 (0.51)	0.34 (0.59)
RF	40–60%	0.65	0.02	0.18 (0.82)	0.32 (0.64)	0.12 (0.95)
RF	60–80%	0.90	0.00	0.09 (0.95)	0.07 (0.99)	0.15 (0.89)
RF	80–100%	0.89	0.00	0.06 (0.99)	0.16 (0.88)	0.11 (0.94)
TA	Before Strike	0.00	0.32	0.88 (0.07)	1.71 (0.00)	0.74 (0.04)
TA	0–20%	0.93	0.00	0.11 (0.92)	0.09 (0.97)	0.06 (0.99)
TA	20–40%	0.48	0.03	0.15 (0.86)	0.40 (0.45)	0.33 (0.76)
TA	40–60%	0.68	0.02	0.22 (0.76)	0.33 (0.71)	0.03 (1.00)
TA	60–80%	0.45	0.03	0.24 (0.67)	0.53 (0.92)	0.36 (0.43)
TA	80–100%	0.42	0.04	0.31 (0.52)	0.27 (0.99)	0.34 (0.46)
GM	Before Strike	0.03	0.13	1.01 (0.03)	0.47 (0.25)	0.41 (0.54)
GM	0–20%	0.00	0.35	1.78 (0.00)	1.17 (0.00)	0.36 (0.59)
GM	20–40%	0.71	0.01	0.19 (0.81)	0.29 (0.71)	0.06 (0.98)
GM	40–60%	0.35	0.04	0.39 (0.42)	0.41 (0.42)	0.00 (1.00)
GM	60–80%	0.28	0.05	0.40 (0.42)	0.49 (0.30)	0.10 (0.97)
GM	80–100%	0.47	0.03	0.24 (0.73)	0.19 (0.89)	0.40 (0.45)
GL	Before Strike	0.50	0.03	0.25 (0.73)	0.43 (0.47)	0.14 (0.91)
GL	0–20%	0.04	0.12	0.30 (0.60)	0.90 (0.04)	0.60 (0.26)
GL	20–40%	0.23	0.06	0.41 (0.36)	0.54 (0.25)	0.10 (0.97)
GL	40–60%	0.04	0.13	0.72 (0.06)	0.70 (0.08)	0.06 (0.99)
GL	60–80%	0.17	0.11	0.47 (0.55)	0.79 (0.15)	0.33 (0.68)
GL	80–100%	0.21	0.06	0.62 (0.18)	0.30 (0.57)	0.32 (0.72)

**Table A2:** ES for time of maximum EMG energy among foot strike patterns during stance phase (related to Figure 2).

Muscle	*p*	η^2^	Cohens’ d (*p* Value)		
			FFS vs. MFS	FFS vs. RFS	MFS vs. RFS
VM	0.00	0.27	0.08 (0.80)	1.72 (0.00)	1.17 (0.00)
VL	0.00	0.44	0.71 (0.02)	2.62 (0.00)	1.29 (0.00)
RF	0.01	0.18	0.65 (0.07)	1.19 (0.00)	0.43 (0.20)
TA	0.09	0.10	0.48 (0.17)	0.81 (0.03)	0.30 (0.41)
GM	0.03	0.16	0.01 (0.65)	0.82 (0.04)	1.12 (0.01)
GL	0.00	0.41	0.79 (0.02)	1.92 (0.00)	1.31 (0.00)

**Table A3:** ES for normalized EMG power comparisons between foot strike patterns and running phases (related to Figure 3).

Muscle	Phase	Frequency Band	*p*	η^2^	Cohens’ d (*p* Value)		
					FFS vs. MFS	FFS vs. RFS	MFS vs. RFS
VM	0–20%	below 60 Hz	0.55	0.03	0.23 (0.55)	0.17 (0.63)	0.37 (0.28)
VL	0–20%	below 60 Hz	0.89	0.01	0.10 (0.76)	0.06 (0.88)	0.17 (0.64)
RF	0–20%	below 60 Hz	0.53	0.03	0.34 (0.34)	0.38 (0.32)	0.02 (0.96)
TA	0–20%	below 60 Hz	0.22	0.07	0.22 (0.50)	0.40 (0.29)	0.65 (0.09)
GM	0–20%	below 60 Hz	0.03	0.17	0.75 (0.07)	1.12 (0.01)	0.35 (0.40)
GL	0–20%	below 60 Hz	0.19	0.07	0.62 (0.07)	0.34 (0.38)	0.34 (0.33)
VM	0–20%	61–200 Hz	0.47	0.03	0.19 (0.61)	0.26 (0.48)	0.41 (0.23)
VL	0–20%	61–200 Hz	0.78	0.01	0.10 (0.76)	0.14 (0.69)	0.26 (0.48)
RF	0–20%	61–200 Hz	0.57	0.02	0.31 (0.38)	0.36 (0.34)	0.02 (0.95)
TA	0–20%	61–200 Hz	0.50	0.03	0.31 (0.35)	0.05 (0.89)	0.41 (0.28)
GM	0–20%	61–200 Hz	0.01	0.17	0.62 (0.11)	1.19 (0.00)	0.45 (0.15)
GL	0–20%	61–200 Hz	0.13	0.09	0.68 (0.04)	0.35 (0.36)	0.41 (0.25)
VM	20–40%	below 60 Hz	0.38	0.04	0.36 (0.30)	0.09 (0.79)	0.49 (0.19)
VL	20–40%	below 60 Hz	0.95	0.00	0.09 (0.79)	0.09 (0.79)	0.00 (0.99)
RF	20–40%	below 60 Hz	0.46	0.03	0.17 (0.65)	0.28 (0.43)	0.42 (0.22)
TA	20–40%	below 60 Hz	0.74	0.01	0.27 (0.44)	0.14 (0.70)	0.14 (0.70)
GM	20–40%	below 60 Hz	0.48	0.03	0.19 (0.57)	0.47 (0.23)	0.22 (0.53)
GL	20–40%	below 60 Hz	0.80	0.01	0.08 (0.81)	0.16 (0.67)	0.24 (0.51)
VM	20–40%	61–200 Hz	0.35	0.05	0.37 (0.29)	0.12 (0.73)	0.52 (0.16)
VL	20–40%	61–200 Hz	0.95	0.00	0.08 (0.82)	0.11 (0.76)	0.03 (0.94)
RF	20–40%	61–200 Hz	0.50	0.03	0.17 (0.66)	0.26 (0.47)	0.40 (0.25)
TA	20–40%	61–200 Hz	0.65	0.02	0.33 (0.36)	0.14 (0.69)	0.19 (0.60)
GM	20–40%	61–200 Hz	0.58	0.02	0.25 (0.47)	0.40 (0.31)	0.10 (0.77)
GL	20–40%	61–200 Hz	0.46	0.03	0.20 (0.55)	0.25 (0.51)	0.44 (0.22)
VM	Before Strike	below 60 Hz	0.52	0.03	0.30 (0.40)	0.36 (0.28)	0.10 (0.80)
VL	Before Strike	below 60 Hz	0.53	0.03	0.33 (0.30)	0.07 (0.85)	0.37 (0.39)
RF	Before Strike	below 60 Hz	0.70	0.02	0.12 (0.74)	0.30 (0.41)	0.18 (0.62)
TA	Before Strike	below 60 Hz	0.18	0.07	0.28 (0.43)	0.71 (0.07)	0.37 (0.28)
GM	Before Strike	below 60 Hz	0.03	0.14	0.94 (0.02)	0.83 (0.03)	0.03 (0.80)
GL	Before Strike	below 60 Hz	0.14	0.08	0.72 (0.05)	0.49 (0.18)	0.21 (0.53)
VM	Before Strike	61–200 Hz	0.58	0.02	0.30 (0.41)	0.32 (0.34)	0.05 (0.90)
VL	Before Strike	61–200 Hz	0.59	0.02	0.30 (0.34)	0.06 (0.87)	0.33 (0.43)
RF	Before Strike	61–200 Hz	0.80	0.01	0.12 (0.74)	0.23 (0.51)	0.12 (0.74)
TA	Before Strike	61–200 Hz	0.63	0.02	0.26 (0.43)	0.35 (0.38)	0.03 (0.93)
GM	Before Strike	61–200 Hz	0.03	0.14	1.03 (0.01)	0.71 (0.05)	0.16 (0.62)
GL	Before Strike	61–200 Hz	0.12	0.09	0.73 (0.05)	0.52 (0.16)	0.21 (0.54)

## References

[1] Daoud A.I., Geissler G.J., Wang F., Saretsky J., Daoud Y.A., Lieberman D.E. (2012). Foot strike and injury rates in endurance runners: A retrospective study. Med. Sci. Sports Exerc..

[2] Xu D., Zhou H., Quan W., Jiang X., Liang M., Li S., Ugbolue U.C., Baker J.S., Gusztav F., Ma X. (2024). A new method proposed for realizing human gait pattern recognition: Inspirations for the application of sports and clinical gait analysis. Gait Posture.

[3] Xu Y., Yuan P., Wang R., Wang D., Liu J., Zhou H. (2021). Effects of Foot Strike Techniques on Running Biomechanics: A Systematic Review and Meta-analysis. Sports Health.

[4] Yang L., Li M., Liu Y., Shi S. (2022). Neuromuscular tuning patterns of Running with Different Landing Methods: Time-frequency Analysis Based on Ground Reaction Forces. J. Sports Sci..

[5] Kuntze G., von Tscharner V., Hutchison C., Ronsky J.L. (2015). Multi-muscle activation strategies during walking in female post-operative total joint replacement patients. J. Electromyogr. Kinesiol..

[6] Huber C., Nuesch C., Gopfert B., Cattin P.C., von Tscharner V. (2011). Muscular timing and inter-muscular coordination in healthy females while walking. J. Neurosci. Methods.

[7] Huber C., Federolf P., Nuesch C., Cattin P.C., Friederich N.F., Tscharner V. (2013). Heel-strike in walking: Assessment of potential sources of intra- and inter-subject variability in the activation patterns of muscles stabilizing the knee joint. J. Biomech..

[8] Gopfert B., Scharer C., Tacchelli L., Gross M., Luthy F., Hubner K. (2022). Frequency Shifts in Muscle Activation during Static Strength Elements on the Rings before and after an Eccentric Training Intervention in Male Gymnasts. J. Funct. Morphol. Kinesiol..

[9] Doorenbosch C.A., Harlaar J., Roebroeck M.E., Lankhorst G.J. (1994). Two strategies of transferring from sit-to-stand; the activation of monoarticular and biarticular muscles. J. Biomech..

[10] Tam N., Santos-Concejero J., Coetzee D.R., Noakes T.D., Tucker R. (2017). Muscle co-activation and its influence on running performance and risk of injury in elite Kenyan runners. J. Sports Sci..

[11] von Tscharner V. (2000). Intensity analysis in time-frequency space of surface myoelectric signals by wavelets of specified resolution. J. Electromyogr. Kinesiol..

[12] Maurer C., von Tscharner V., Nigg B.M. (2013). Speed-dependent variation in the Piper rhythm. J. Electromyogr. Kinesiol..

[13] Jaitner T., Janssen D., Burger R., Wenzel U. Identification of EMG frequency patterns in running by wavelet analysis and support vector machines. Proceedings of the ISBS Conference Proceedings Archive.

[14] Al-Mulla M.R., Sepulveda F., Colley M. (2011). A Review of Non-Invasive Techniques to Detect and Predict Localised Muscle Fatigue. Sensors.

[15] Chowdhury R.H., Reaz M.B., Ali M.A., Bakar A.A., Chellappan K., Chang T.G. (2013). Surface electromyography signal processing and classification techniques. Sensors.

[16] Huang N.E., Shen Z., Long S.R., Wu M.C., Shih H.H., Zheng Q., Yen N.-C., Tung C.C., Liu H.H. (1998). The empirical mode decomposition and the Hilbert spectrum for nonlinear and non-stationary time series analysis. Proc. R. Soc. Lond. Ser. A Math. Phys. Eng. Sci..

[17] Andrade A.O., Nasuto S., Kyberd P., Sweeney-Reed C.M., Van Kanijn F.R. (2006). EMG signal filtering based on Empirical Mode Decomposition. Biomed. Signal Process. Control.

[18] Sun Z., Xi X., Yuan C., Yang Y., Hua X. (2020). Surface electromyography signal denoising via EEMD and improved wavelet thresholds. Math. Biosci. Eng..

[19] Zhang X., Zhou P. (2013). Filtering of surface EMG using ensemble empirical mode decomposition. Med. Eng. Phys..

[20] Chang K.M., Liu S.H., Wang J.J., Cheng D.C. (2013). Exercise muscle fatigue detection system implementation via wireless surface electromyography and empirical mode decomposition. Annu. Int. Conf. IEEE Eng. Med. Biol. Soc..

[21] Cruz-Montecinos C., Garcia-Masso X., Maas H., Cerda M., Ruiz-Del-Solar J., Tapia C. (2023). Detection of intermuscular coordination based on the causality of empirical mode decomposition. Med. Biol. Eng. Comput..

[22] Molinari F., Joy Martis R., Acharya U.R., Meiburger K.M., De Luca R., Petraroli G., Liboni W. (2015). Empirical mode decomposition analysis of near-infrared spectroscopy muscular signals to assess the effect of physical activity in type 2 diabetic patients. Comput. Biol. Med..

[23] Liu S.-H., Chang K.-M., Cheng D.-C. (2014). The progression of muscle fatigue during exercise estimation with the aid of high-frequency component parameters derived from ensemble empirical mode decomposition. IEEE J. Biomed. Health Inform..

[24] Li Y., Li J., Tu P., Wang H., Wang K. (2023). Gesture recognition based on EEMD and cosine Laplacian eigenmap. IEEE Sens. J..

[25] Hopkins W.G. (2000). Measures of reliability in sports medicine and science. Sports Med..

[26] Brysbaert M. (2019). How many participants do we have to include in properly powered experiments? A tutorial of power analysis with reference tables. J. Cogn..

[27] Mesquida C., Murphy J., Lakens D., Warne J. (2023). Publication bias, statistical power and reporting practices in the Journal of Sports Sciences: Potential barriers to replicability. J. Sports Sci..

[28] Hermens H.J., Freriks B., Merletti R., Stegeman D., Blok J., Rau G., Disselhorst-Klug C., Hägg G. (1999). European recommendations for surface electromyography. Roessingh Res. Dev..

[29] Hasegawa H., Yamauchi T., Kraemer W.J. (2007). Foot strike patterns of runners at the 15-km point during an elite-level half marathon. J. Strength Cond. Res..

[30] Xu D., Zhou H., Quan W., Ma X., Chon T.-E., Fernandez J., Gusztav F., Kovács A., Baker J.S., Gu Y. (2024). New insights optimize landing strategies to reduce lower limb injury risk. Cyborg Bionic Syst..

[31] Martinek R., Ladrova M., Sidikova M., Jaros R., Behbehani K., Kahankova R., Kawala-Sterniuk A. (2021). Advanced Bioelectrical Signal Processing Methods: Past, Present, and Future Approach-Part III: Other Biosignals. Sensors.

[32] Kiewiet H., Bulsink V.E., Beugels F., Koopman H. (2017). The co-contraction index of the upper limb for young and old adult cyclists. Accid. Anal. Prev..

[33] Landreneau L.L., Watts K., Heitzman J.E., Childers W.L. (2014). Lower limb muscle activity during forefoot and rearfoot strike running techniques. Int. J. Sports Phys. Ther..

[34] Valencia O., Weinstein A., Salas R., Guzman-Venegas R., Arvanitidis M., Martinez-Valdes E. (2023). Temporal differences in the myoelectric activity of lower limb muscles during rearfoot and forefoot running: A statistical parametric mapping approach. Eur. J. Sport Sci..

[35] Olin E.D., Gutierrez G.M. (2013). EMG and tibial shock upon the first attempt at barefoot running. Hum. Mov. Sci..

[36] von Tscharner V. (2019). A model computation of how synchronization and clustering of motor unit action potentials alter the power spectra of electromyograms. Biomed. Signal Process. Control.

[37] Koenig I., Eichelberger P., Blasimann A., Hauswirth A., Baeyens J.P., Radlinger L. (2018). Wavelet analyses of electromyographic signals derived from lower extremity muscles while walking or running: A systematic review. PLoS ONE.

[38] von Tscharner V., Nigg B.M. (2008). Last word on point:counterpoint: Spectral properties of the surface EMG can characterize/do not provide information about motor unit recruitment strategies and muscle fiber type. J. Appl. Physiol..

[39] Yong J.R., Dembia C.L., Silder A., Jackson R.W., Fredericson M., Delp S.L. (2020). Foot strike pattern during running alters muscle-tendon dynamics of the gastrocnemius and the soleus. Sci. Rep..

[40] von Tscharner V., Ullrich M., Mohr M., Comaduran Marquez D., Nigg B.M. (2018). A wavelet based time frequency analysis of electromyograms to group steps of runners into clusters that contain similar muscle activation patterns. PLoS ONE.

[41] von Tscharner V., Barandun M., Stirling L.M. (2011). Piper rhythm of the electromyograms of the abductor pollicis brevis muscle during isometric contractions. J. Electromyogr. Kinesiol..

[42] Latash M.L. (2018). Muscle coactivation: Definitions, mechanisms, and functions. J. Neurophysiol..

[43] Tam N., Tucker R., Santos-Concejero J., Prins D., Lamberts R.P. (2019). Running Economy: Neuromuscular and Joint Stiffness Contributions in Trained Runners. Int. J. Sports Physiol. Perform..

